# Intracystic Hemorrhage and Its Management During Ultrasound-Guided Percutaneous Microwave Ablation for Cystic Thyroid Nodules

**DOI:** 10.3389/fendo.2020.00477

**Published:** 2020-09-08

**Authors:** Su Dong, Lele Sun, Jialu Xu, Zhe Han, Jia Liu

**Affiliations:** ^1^Department of Anesthesia, The First Hospital of Jilin University, Changchun, China; ^2^Department of Thyroid Surgery, The First Hospital of Jilin University, Changchun, China

**Keywords:** intracystic hemorrhage, microwave ablation, cystic thyroid nodule, complications, ultrasound

## Abstract

**Background:** Intracystic hemorrhage can present occasionally during ultrasound-guided percutaneous microwave ablation (PMWA) for cystic thyroid nodules. It can affect treatment outcome, can lead to ablation failure, and even conversion to open surgery. We aim to avoid such cases in the future by exploring their causes and management.

**Methods:** From March 2017 to December 2019, we retrospectively studied 87 cystic thyroid nodules in 59 patients who underwent PMWA in the First Hospital of Jilin University. All patients were followed at 1, 3, 6, and 12 months after treatment.

**Results:** All patients completed the treatment successfully. Nine cystic thyroid nodules presented with intracystic hemorrhage during the ultrasound-guided PMWA, giving an incidence of 10.3% (9/87 cysts). Larger cystic thyroid nodules were more likely to develop intracystic bleeding during ultrasound-guided PMWA. Intracystic hemorrhage resulted in significantly prolonged ablation time and had a negative effect on treatment outcome. No patients had other complications, but temporary post-operative pain and local swelling were more obvious in patients with intracystic hemorrhage.

**Conclusion:** Intracystic hemorrhage is not rare during ultrasound-guided PMWA for cystic thyroid nodules. Doctors should pay more attention to it, learn to manage it and try to avoid it in clinical practice.

## Introduction

Recent developments in imaging techniques have led to an increase in diagnosis of thyroid nodules (1). Many of these nodules are pure cystic or predominantly cystic (cystic component ≥60%). According to the guidelines of the American Thyroid Association (2), cystic nodules are considered to be of negligible malignancy risk (pure cysts, <1%; partially cystic nodules with no suspicious features, <3%; partially cystic nodules with eccentric solid areas with low suspicious features, 5–10%). However, cystic thyroid nodules still require treatment in some cases, such as those with rapid growth, aesthetic disturbances, or local compressive symptoms (neck pain, dysphagia, and cough), or in patients who are worried (3). At present, treatment options include simple nodule aspiration, ablation, or surgical resection (4). Aspiration can be performed easily, but recurrence rates are high, observed in 60–90% of patients (5). Surgical treatment is more definitive but may be associated with scarring and more complications. Ablation has been increasingly accepted as the best treatment for cystic thyroid nodules (6). Currently, the following types of ablation therapies are used to treat thyroid cystic nodules: ethanol ablation, laser ablation, radiofrequency thermal ablation, and microwave ablation (MWA) (7).

MWA is a new local thermal ablation technique that has a fast heating speed, strong coagulation ability, and a large ablation zone, so this technique has become a major method in heat ablation therapy. It is an effective treatment of thyroid nodules worldwide (8–11). We found that intracystic hemorrhage appeared occasionally when ultrasound-guided PMWA was used to treat cystic thyroid nodule, and interfered with treatment. However, to date there has been no study focusing and reporting on such issues.

Our study is believed to be the first to analyze the causes of intracystic hemorrhage and explore the corresponding solutions during ultrasound-guided PMWA for cystic thyroid nodules. We aimed to avoid intracystic hemorrhage and improve the efficacy of ultrasound-guided PMWA to treat cystic thyroid nodules.

## Materials and Methods

### Patients

A total of 87 cystic thyroid nodules in 59 patients (47 female and 12 male; mean age 48.7 ± 1.1 years; range: 18–78 years) were treated with ultrasound-guided PMWA between March 2017 and December 2019 in the First Hospital of Jilin University (Table 1). All nodules were examined through pre-operative or post-operative ultrasound-guided fine-needle aspiration (FNA) cytology and were confirmed as benign pathologically. The 59 patients with one or more pure cystic nodules or predominantly cystic nodules (cystic component ≥60%) were selected based on the following inclusion criteria: (1) >50% increased cystic volume in 3 months; (2) anxiety about thyroid nodules and willingness to undergo treatment; (3) presence of subjective symptoms such as neck discomfort or pain, or difficulty in breathing or swallowing; and (4) overall poor condition that could not tolerate surgery, or unwillingness to undergo surgery for aesthetic reasons. The exclusion criteria included: (1) coagulation dysfunction, severe bleeding tendency, severe cardiopulmonary disease that could not tolerate treatment; and (2) FNA results that indicated follicular or malignant tumors.

**Table 1:** Characteristics of patients with cystic thyroid nodules.

**Characteristics**	**All patients (n = 59)**	**After matching**		
		**Bleeding group (n = 9)**	**Control group (n = 11)**	***p*-value**
Female/Male	47/12	8/1	8/3	0.369
Age (y)	48.7 ± 1.1	43.8 ± 4.1	42.7 ± 3.7	0.333
Nodule diameter(cm)	2.82 ± 1.12	3.93 ± 0.87	3.42 ± 0.39	0.096
Nodule volume (ml)	18.32 ± 1.78	21.06 ± 1.75	20.01 ± 1.45	0.159

*Except for p-values, data are reported as mean ± standard deviations*.

### Equipment

The GE logiq E9 ultrasound diagnostic device with a line array probe (frequency 6–15 MHz) was used. MWA was performed using Nanjing Yigao ECO-100 multifunctional microwave therapeutic instrument with a disposable microwave ablation needle antenna (16G). The antenna type (10 cm long, 1.6 mm diameter, and 3 mm long active tip) was suitable for superficial organs. The output power setting was 30 W, with a frequency of 2450 MHz, and the internally cooled needle antenna with normal saline for cold circulation fluid was used.

### Ablation Procedure

The best puncture path and ablation were determined based on the size, location, and blood flow of the thyroid cystic nodules. During ablation, the patient was placed in the supine position with a fully exposed neck. Lidocaine (2%) (5 ml) was infused into the surrounding thyroid capsule to reduce pain stimulation when performing the puncture needle and heat ablation under ultrasound guidance before ablation. A 20 ml syringe was used to aspirate the internal fluid of the nodules before MWA. Saline (0.9%) was used to dilute cystic fluid to facilitate suction. MWA was performed when little fluid remained in the nodule and the volume of the nodule was significantly reduced. Under ultrasound guidance, the microwave needle was pinned accurately into the nodules and the MWA treatment was started, with a power output of 25–40 W. The ablation process was carried out under ultrasonic dynamic monitoring. The area adjacent to the capsule wall and the solid part of the nodules were paid more attention during MWA. When the multi-angle scanning showed a strong change of echo after ablation and when color Doppler flow imaging (CDFI) showed no blood flow signal in the nodules, ablation was completed. All patients were kept under observation for >2 h after treatment, with local compression of the neck lasting 30 min.

### Follow-Up

The operation time, post-operative hospitalization and post-operative complication rates were recorded. The symptomatic scores in patients were self-measured by the patients with a 10 cm visual analog scale (0–10). Ultrasound assessments were performed at 1, 3, 6, and 12 months after treatment. The size and echogenicity of the nodules were examined, and the volume calculated. The volume reduction rate (VRR) was calculated using the following equation: VRR (%) = (pre-treatment volume-follow-up volume)/pre-treatment volume × 100%. Thyroid function (tri-iodothyronine, thyroxine, and thyroid-stimulating hormone) was also examined during the follow-up period.

### Statistical Analysis

SPSS version 20.0 was used for data analyses. Quantitative data were expressed as mean ± standard deviation. The two groups were compared using the independent samples *t*-test. The number of cases or percentages of numerical data were expressed and comparison between the two groups was performed using the χ^2^-test. Quantitative data were compared between the two groups by the Mann–Whitney *U*-test. The follow-up nodule volume and VRR of the nodule after MWA were compared with baseline volume by means of the Wilcoxon tests. *P* < 0.05 was considered statistically significant.

## Results

### Incidence of Intracystic Hemorrhage and Its Influencing Factors

**Figure 1:**
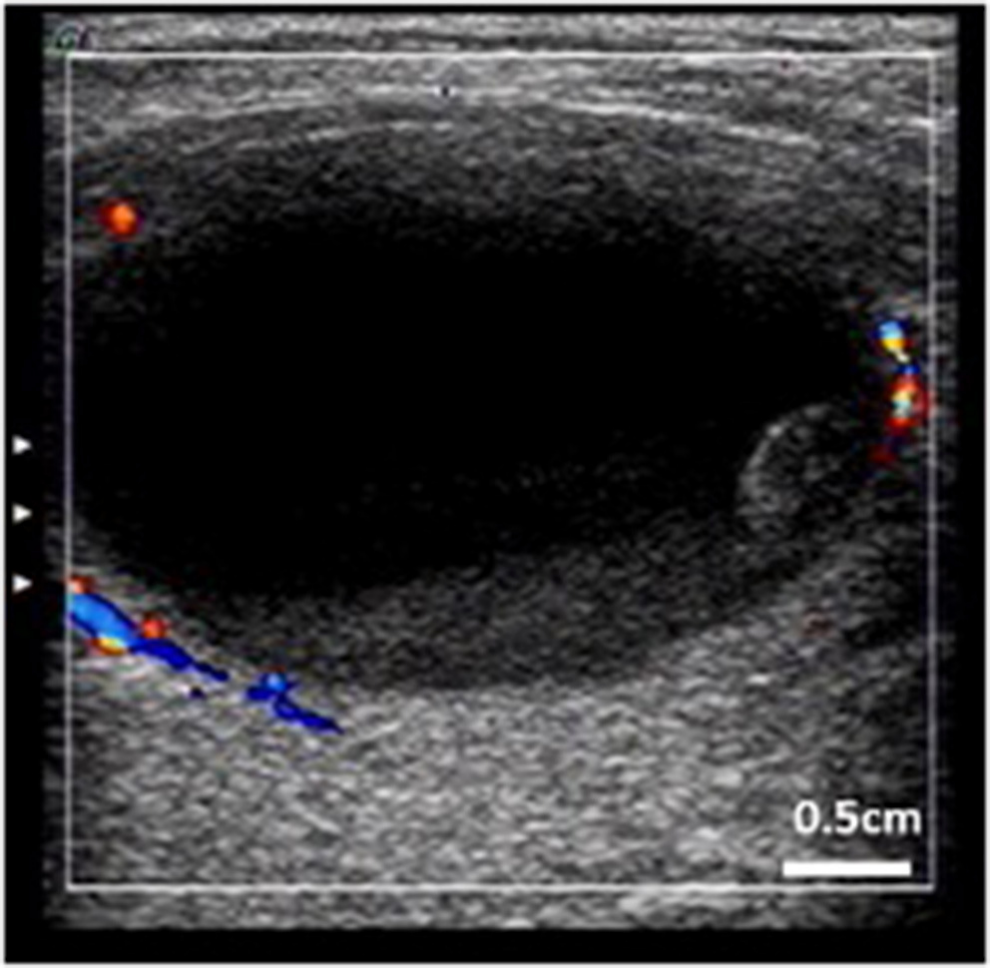
Thyroid cystic nodule prone to intracystic hemorrhage. (Characteristic: bigger, more cystic components, peripheral blood flow, and stale hemorrhage in the nodule).

Nine cystic thyroid nodules in nine patients presented with intracystic hemorrhage during ultrasound-guided PMWA, giving an incidence of 10.3% (9/87 cysts); 77.8% of intracystic hemorrhage (7/9 cysts) occurred in women aged 40–60 years (Table 2). Intracystic hemorrhage occurred in nodules measuring 3.1–4.8 cm (mean size 3.9 cm) (Table 1). All intracystic hemorrhage occurred in pure cysts or those with cystic components >80%. Middle-aged women with larger nodules were more prone to intracystic bleeding (Table 2, Figure 1).

**Table 2:** Risk factors of intracystic hemorrhage with cystic thyroid nodules (n).

**Group**	**Female/Male**	**Female**		**Nodule diameter**	
		40–60 y	>60/<40 y	≥3.5 cm	<3.5 cm
Bleeding (*n* = 9)	8/1	7	1	6	3
No-Bleeding (*n* = 50)	39/11	18	21	11	39
*χ^2^*	0.558	4.558		7.491	
*p*-value	0.455	0.033		0.006	

### Management of Intracystic Hemorrhage During Ultrasound-Guided PMWA

**Figure 2:**
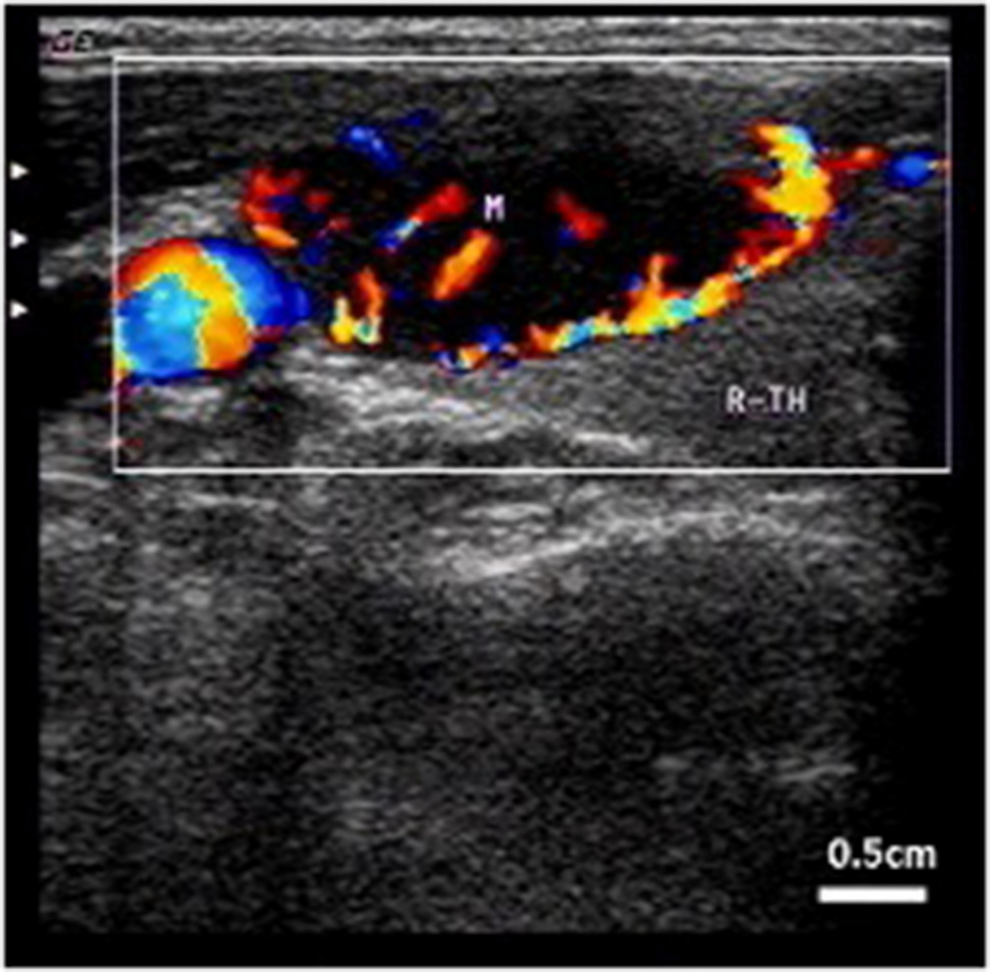
Intracystic hemorrhage. Color blood flow can be observed by CDFI. Arrow indicates internal carotid artery.

Intracystic hemorrhage occurred after aspiration of cyst fluid and before MWA in most cases (7/9, 77.8%). The nodules that had shrunk gradually enlarged again under ultrasound monitoring. Sometimes the enlargement was visible to the naked eye, and in some special cases, bleeding points and blood flow could be seen. Fresh blood was found when sucked again. Bleeding was confined to the cysts and did not affect other tissues. When intracystic hemorrhage occurred, the operator stopped the procedure. Local compression was used to stop the bleeding for 3–5 min. If the bleeding stopped, the previous aspiration and ablation steps were repeated. If bleeding persisted or recurred after the procedure, a 20 ml syringe and ablation needle were placed into the cyst at the same time. Aspiration, observation, searching for the bleeding point and ablation were performed simultaneously. Treatment of possible bleeding sites near the cyst capsule should be emphasized. The hemorrhagic vessels were closed by heat ablation. All cases of intracystic hemorrhage were stopped in the first place. Intracystic hematomas formed locally when several cases took too long to treat (Figure 2).

### Intracystic Hemorrhage Increased Operation Time

All cases of intracystic hemorrhage were classified as the bleeding group (*n* = 9). Eleven patients, whose basic characteristics were matched, were chosen from the remaining cases as the control group (Table 1). The operation time for each nodule is summarized in Table 3. In the bleeding group, the operation time for each nodule was 48.6 ± 12.2 min. In the control group, the operation time was 25.6 ± 6.2 min (*p* < 0.05). Intracystic hemorrhage increased the complexity of the operation and the operation time for each nodule was significantly longer.

**Table 3:** Comparison of several indicators between two groups.

	**Bleeding group**	**Control group**	***p*-value**
Operation time (min)	48.6 ± 12.2	25.6 ± 6.2	0.013
Symptomatic score-1 d	5.2 ± 1.3	3.2 ± 1.1	0.032
Symptomatic score-3 d	2.1 ± 0.7	1.3 ± 0.3	0.107
Hospital stay (d)	2.3 ± 1.0	1.0 ± 0.3	0.000

*Except for p-values, data are reported as mean ± standard deviations*.

### Intracystic Hemorrhage Caused More Significant Post-operative Symptoms and Extended Hospital Stay

The mean symptomatic score of the bleeding group (5.2 ± 1.3) was higher than in the control group (3.2 ± 1.1) at 1 day after the operation (*p* < 0.05) (Table 3). The most common symptoms were pain and swelling. There was no significant difference in mean symptomatic scores between the two groups (2.1 ± 0.7 and 1.3 ± 0.3) at 3 days after the operation. In the bleeding group, the mean hospital stay was 2.3 ± 1.0 days. In the control group, the mean hospital stay was 1.0 ± 0.3 days (*p* < 0.01).

### Intracystic Hemorrhage Affected Treatment Effectiveness of MWA for Cystic Thyroid Nodules

The average volume of the bleeding group cysts was 21.06 ± 1.75 ml before treatment, 16.49 ± 1.83 ml at 1 month, 12.24 ± 1.54 ml at 3 months, 10.61 ± 1.66 ml at 6 months, and 9.14 ± 1.56 ml at 12 months after the operation. The VRR gradually increased by 21.7% at 1 month, 41.9% at 3 months, 49.6% at 6 months, and 56.6% at 12 months. For the control group, the average cyst volume was 20.01 ± 1.45 ml before treatment, 10.18 ± 1.13 ml at 1 month, 7.21 ± 0.88 ml at 3 months, 4.21 ± 0.63 ml at 6 months, and 3.24 ± 0.51 ml at 12 months. The VRR gradually increased by 49.1% at 1 month, 64.0% at 3 months, 79.0% at 6 months, and 83.8% at 12 months. The VRR of the bleeding group was significantly lower than that of the control group (*p* < 0.01) (Table 4). All patients in the control group were satisfied with the treatment effect. One patient in the bleeding group was treated again with MWA after 12 months due to unsatisfactory treatment. No other serious complications were observed in either group.

**Table 4:** Treatment effectiveness of MWA for cystic thyroid nodules.

**Time**	**Bleeding group**		**Control group**		***p*-value**
	**Volume (ml)**	**VRR (%)**	**Volume (ml)**	**VRR (%)**	
Before MWA	21.06 ± 1.75	–	20.01 ± 1.45	–	–
1 month after MWA	16.49 ± 1.83	21.7	10.18 ± 1.13	49.1	0.000
3 months after MWA	12.24 ± 1.54	41.9	7.21 ± 0.88	64	0.000
6 months after MWA	10.61 ± 1.66	49.6	4.21 ± 0.63	79	0.000
12 months after MWA	9.14 ± 1.56	56.6	3.24 ± 0.51	83.8	0.000

*Volume was expressed by the mean ± standard deviation. VRR: volume reduction rate*.

## Discussion

Many studies have shown that ultrasound-guided MWA is an effective and safe technique for the treatment of benign thyroid nodules, including cystic and solid nodules. Ultrasound-guided MWA can significantly reduce nodule volume, improve clinical symptoms, cause fewer complication, guarantee quick recovery, meet patients' aesthetic needs, and show less physiological and psychological interference (11–13). Therefore, it is being increasingly used clinically. In our practice, we found that intracystic hemorrhage occurred occasionally when we treated thyroid cystic nodules with MWA. Intracystic hemorrhage interfered with the treatment and had an adverse impact on the eventual efficacy. So, clinicians should pay more attention to and try to avoid intracystic hemorrhage.

We analyzed the causes of intracystic hemorrhage. There was intracystic hemorrhage originally, and the bleeding stopped as the pressure increased due to bleeding in the closed capsule. The ablation treatment time was too soon after the original bleeding time. During the ablation process, the cyst fluid was suctioned and local compression relieved. These caused the original bleeding site to bleed again. There were many large blood vessels around the cyst. The needle easily damaged the blood vessels and caused bleeding when cysts shrank by puncturing and suction of cyst fluid (3). Improper procedures or inadequate skill or experience of the operators were likely to cause intracystic hemorrhage. In order to avoid intracystic hemorrhage, we should do the following. (1) Before using MWA to treat cystic thyroid nodules, a detailed history should be taken, including recent changes in the size of the nodules, with or without tenderness. MWA should be postponed if there was recent intracystic bleeding. The ideal treatment time is 3–4 weeks after the anterior neck pain disappears. (2) The needle should be as far away from the capsule wall as possible when puncturing and aspirating cyst fluid. Operators need to adjust the needle position at any time during aspiration of the cyst fluid and the cyst shrinks. (3) CDFI should be used to observe carefully the blood flow around the cyst before ablation. Large blood vessels should be avoided in advance. (4) Operators should be proficient in basic techniques of ablation, such as the moving shot technique, the multiple overlapping shot technique, the hydrodissection technique, and leveraged pry-off method (14–17). Insufficient experience or unskilled operation can easily lead to failure of treatment, and even the risk of uncontrollable bleeding that requires conversion to open surgery.

In actual practice, we have encountered such a situation. The volume of cystic nodules significantly reduced after aspiration of cystic fluid but the cyst volume increased again after 3–5 s, and the size even exceeded that before treatment. Fresh blood was found by aspirating again. Local compression stopped cystic bleeding temporarily, but bleeding persisted after decompression. It is important not to panic in this situation and calm the mood of the patient. Operators can place a 20 ml syringe with needle and the ablation antenna into the cyst together. Aspiration, observation, searching for the bleeding point and ablation are performed simultaneously. The localized high temperature generated by MWA is used to close the bleeding point for the first time. It should be noted that the operation must be performed quickly and accurately. The intracapsular hemorrhage will form a hematoma if too much time is taken to deal with bleeding, which will affect the treatment outcome. There is no need to suck out all the cystic fluid. Operators should be careful to ensure safety and to prevent other complications caused by rushed operations.

Currently, some reports still recommended ethanol ablation as a first-line treatment for predominantly thyroid cystic nodules (18). In our study, the cyst volume gradually reduced after MWA. Compared to the baseline, MWA can achieve nearly 84% of the volume reduction and improve the clinical symptoms. Meanwhile, the adverse effects are negligible. So according to our experiences and other research results, we thought that MWA for thyroid cystic nodules achieved good results, and relapse was difficult.

Our study had some limitations. First, there was a limited number of patients. We will increase the number of cases in our future work. Second, follow-up time was short for a complete research cycle. This will be improved in the next study.

## Conclusion

Our study revealed the causes of intracystic hemorrhage and explored ultrasound-guided PMWA for cystic thyroid nodules. We hope that our report will help surgeons take precautionary measures to minimize the possibility of intracystic hemorrhage. This will make MWA more effective for clinical treatment of thyroid cysts.

## Data Availability Statement

The raw data supporting the conclusions of this article will be made available by the authors, without undue reservation, to any qualified researcher.

## Ethics Statement

The studies involving human participants were reviewed and approved by Ethics committee of First Hospital of Jilin University. The patients/participants provided their written informed consent to participate in this study. Written informed consent was obtained from the individual(s) for the publication of any potentially identifiable images or data included in this article.

## Author Contributions

SD wrote the manuscript. LS and JX collected the data. ZH did the statistical analysis. JL designed the protocol. All authors contributed to the article and approved the submitted version.

## Conflict of Interest

The authors declare that the research was conducted in the absence of any commercial or financial relationships that could be construed as a potential conflict of interest.
